# Targeting purinergic pathway to enhance radiotherapy-induced immunogenic cancer cell death

**DOI:** 10.1186/s13046-022-02430-1

**Published:** 2022-07-15

**Authors:** Xuhui Bao, Liyi Xie

**Affiliations:** 1 Institute of Therapeutic Cancer Vaccines, Fudan University Pudong Medical Center, 2800 Gongwei Rd, Shanghai, China; 2 Department of Oncology, Fudan University Pudong Medical Center, Shanghai, China; 3 Department of Pathology, Duke University Medical Center, Durham, NC USA; 4 Department of Radiation Oncology, Fudan University Shanghai Cancer Center, 270 Dong An Rd, Shanghai, China; 5 Department of Oncology, Shanghai Medical College, Fudan University, Shanghai, China

**Keywords:** Purinergic pathway, Radiotherapy, Immunogenic cell death, Immunotherapy, Cancer

## Abstract

Emerging evidence has demonstrated that radiotherapy (RT) can not only cause direct damage to cancer cells but also lead to immunogenic cell death (ICD), which involves the activation of host antitumor immune response in tumor immune microenvironment (TIME). RT-induced ICD comprises the release of damage-associated molecular patterns (DAMPs) from dying cancer cells that result in the activation of tumor-specific immunity to elicit long-term antitumor efficacy in both original and abscopal tumor sites. Adenosine triphosphate (ATP), as an important DAMP released by irradiated cancer cells and an essential factor within purinergic pathway, can be further hydrolyzed to adenosine (ADO) by two key ectonucleotidases, CD39 and CD73, to further modulate the antitumor immunity in TIME through purinergic signaling via the interaction to its specific receptors such as adenosine 2A receptor (A2AR) and A2BR widely expressed on the surface of the components in TIME, including cancer cells and many immune effector cells. In this review, we first introduced key components in purinergic pathway including ATP, ADO, their receptors, and essential ectonucleotidases. Then we reviewed the regulation of ATP and ADO levels and their main mechanisms by which they promote tumor growth and broadly suppress antitumor immunity through inhibiting the pro-inflammatory response of dendritic cells, cytotoxic T lymphocytes, and natural killer cells, while improving the anti-inflammatory response of regulatory T cells, macrophages, and myeloid-derived suppressor cells in TIME, especially after irradiation. Finally, we presented an overview of dozens of promising therapeutics including pharmacological antagonists and specific antibodies targeting ADO receptors and ectonucleotidases CD39 or CD73 investigated in the clinic for cancer treatment, especially focusing on the preclinical studies and clinical trials being explored for blocking the purinergic signaling to enhance RT as a combination antitumor therapeutic strategy, which has a robust potential to be translated to the clinic in the future.

## Background

### Brief introduction of purinergic signaling pathway

In 1970s, Burnstock, G. and colleagues introduced their finding that adenosine triphosphate (ATP) might be the neurotransmitter involved in both gut and bladder, which initiated the concept and further exploration of purinergic signaling pathway, i.e., extracellular nucleotides signaling [1]. Early studies largely on the biochemistry, physiology, and pharmacology of purinergic signaling pathway revealed its important functions to transmit signals in nervous system and modulate non-neuronal tissues including endothelial, immune, and inflammatory cells [2]. For example, ATP can serve a dual role in the neuro-immune interaction as both neurotransmitter and immunomodulator. ATP can not only relay the signals invoked by resident immune cells including macrophages and T cells (through the release of cytokines, chemokines, or growth factors) that sensed by peripheral nerves and spinal cord to the brain, but also be released by peripheral sensory afferent neurons to modulate local immune cells including macrophages and T cells to maintain their invoked signals to stimulate peripheral nerves [3–5]. Nowadays, it is well known that ATP, adenosine diphosphate (ADP), uridine triphosphate (UTP), uridine diphosphate (UDP), and adenosine (ADO) are important cellular messengers from purinergic pathway, which modulate various other signaling pathways and participate in numerous physiological and pathological processes, mainly through specific purinergic receptors [6].

### Classification of purinergic receptors

Purinergic receptors have been classified into two families: P1 (sensitive to ADO) and P2 (sensitive to adenine and uridine nucleotides) receptors [6, 7]. P1 receptors (ADO receptors) belong to the G-protein coupled receptor (GPCR) superfamily, which are designated as adenosine 1 receptor (A1R), A2AR, A2BR and A3R. A1R and A3R are mainly coupled to the Gi/o subunit to inhibit adenylate cyclase (AC) and cyclic adenosine monophosphate (cAMP) production, while A2AR and A2BR are mainly coupled to the Gs subunit to increase cAMP synthesis by AC activation [6]. P2 receptors can be further divided into two groups. The first group is P2X receptors (P2XR), which are ligand-gated cation channels receptors (P2X1-P2X7) with ATP as the natural ligand. When activated, P2XRs promote rapid depolarization associated with influx of Ca^2+^ and Na^+^ while efflux of K^+^ [8]. The second group is P2Y receptors (P2YR), which are GPCRs including eight subtypes recognized in mammalian cells: P2Y1, P2Y2, P2Y4, P2Y6 and P2Y11-14. P2YRs can be activated by ATP (P2Y2 and P2Y11), ADP (P2Y1, P2Y12 and P2Y13), UTP (P2Y2 and P2Y4), UDP (P2Y6) and UDP-glucose (P2Y14). P2YRs are coupled via G-proteins (Gq/11, Gs, or Gi/o) to mobilize Ca^2+^, generate/inhibit cAMP, and stimulate the extracellular signal-regulated kinase 1/2 (ERK) / mitogen-activated protein kinase (MAPK) pathway [9]. For instance, recent studies have revealed that P2X7, P2Y1, and P2Y2 play an important role in immunomodulation and inflammation that contributes to neuroinflammatory disorders including Alzheimer’s Disease, Parkinson’s Disease, and multiple sclerosis [2].

### Hydrolysis of nucleotides by ectonucleotidases

**Fig. 1:**
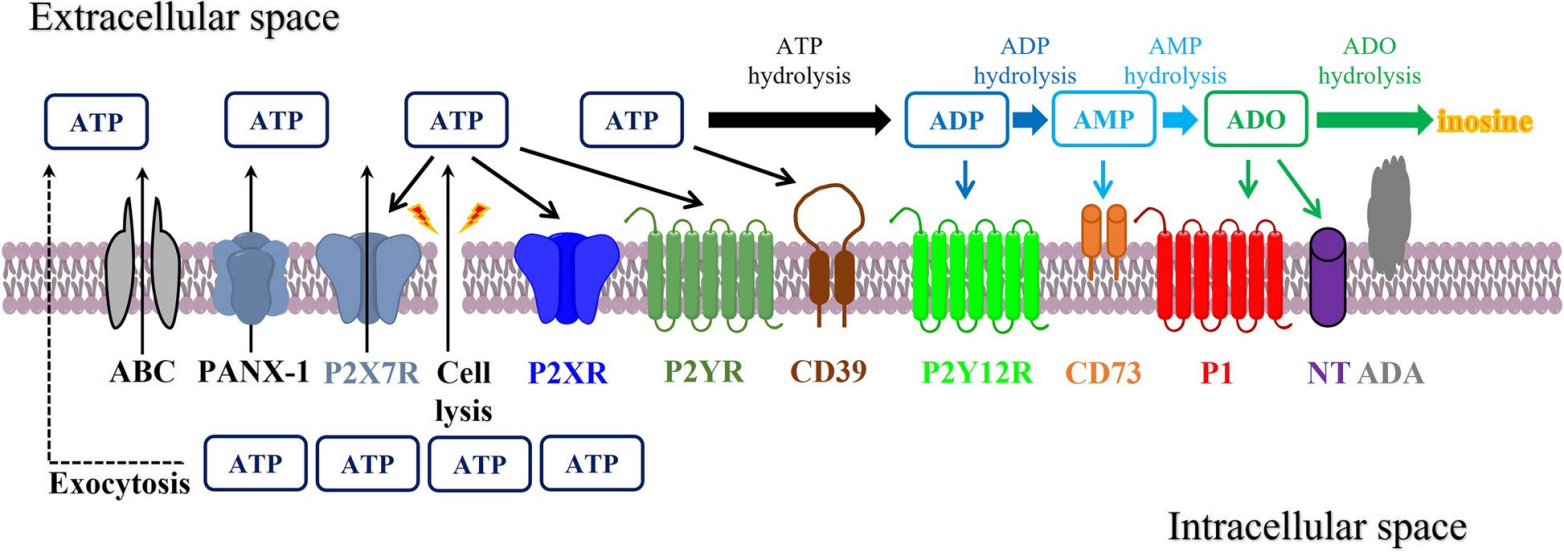
ATP release, receptors, and ectonucleotidases involved in purinergic signaling pathway. ATP is released into the extracellular space via exocytosis, transporters (for example, ABC), channels (for example, PANX-1), P2X7R, or cell lysis (for example, caused by irradiation). Once in the extracellular space, ATP acts at P2XR and P2YR, and is also hydrolyzed to ADP and AMP by ENTPDases such as CD39. ADP can activate P2Y12R and is hydrolyzed to AMP, which can be further hydrolyzed to ADO by CD73. CD73-generated ADO can bind to its P1 receptors (A1R, A2AR, A2BR, and A3R). ADO can be subsequently degraded to inosine by ADA, or transported into the intracellular space via NT. ABC, ATP-binding cassette; ADA, adenosine deaminase; ADO, adenosine; ADP, adenosine diphosphate; AMP, adenosine monophosphate; ATP, adenosine triphosphate; NT, nucleoside transporter; PANX-1, pannexin 1

Once in the extracellular space, ATP can either activate P2R or be further dephosphorylated/hydrolyzed by ectonucleotidases, which have four families: ectonucleoside triphosphate diphosphohydrolases (NTPDases, e.g. CD39/NTPDase 1), ecto-5’-nucleotidase (CD73/NT5E), ectonucleotide pyrophosphatase/phosphodiesterase (ENPP), and alkaline phosphatase (ALP) [10]. Besides limiting ATP signaling, these enzymes can also lead to the generation of additional ligands for P2YRs, for example, ADP to P2Y12, and ADO to A2AR [11]. At the end, ADO can either be hydrolyzed by adenosine deaminase (ADA) to inosine or transported intracellularly by nucleoside transporters (NT) [11] (Fig. 1). Purinergic pathway initiates its function by releasing ATP through paracrine and/or autocrine. Its hydrolysis can subsequently generate a cascade of additional signaling molecules including ADP and ADO. Almost every cell type expresses a different combination of purinergic receptors and ectonucleotidases to regulate this pathway. Hence, the comprehensive effect of purinergic pathway on cellular function depends on not only those specific receptors and ectonucleotidases expressed by the cell, but also the dynamic change of extracellular and intracellular concentrations of ATP and ADO [6, 7].

## Purinergic signaling in tumor immune microenvironment (TIME)

### ATP in TIME

It is well known in previous studies that both ATP and ADO are strong modulators of tumorigenesis and antitumor immune response in TIME, where ectonucleotidases CD39 and CD73 play a fundamental role in modulating the level of ATP and ADO [12–14]. The activation of purinergic receptors is accordingly observed in the setting of inflammation and hypoxia in TIME [13, 15]. First of all, since basic supply of ATP is essential for cancer cell proliferation and tumor growth, TIME contains an elevated level of extracellular ATP comparing to the normal tissue. For example, ATP was within a low nM range in normal tissue, but increased to high µM or mM level in TIME [16]. It has been observed in various lung cancer cell lines that stimulation with high concentrations of ATP (e.g. 0.5–1 mM) favored cancer cell migration and invasion, while ATP in prostate cancer cells can promote the activation of Cdc42 and Rac1 and the expression of matrix metalloproteinases (MMPs) through P2YR activation [17–19]. ATP can also favor tumorigenicity by helping tumors escape from host immunosurveillance. Previous studies revealed that regulatory T cells (Tregs) can be activated at higher concentration of extracellular ATP around 1 mM with tighter cell contact, impeding the activation, expansion, and homing of cytotoxic T lymphocytes (CTL) and T helper cells (T_H_ cells) [20, 21]. In addition, AMP can impede the maturation of dendritic cells (DCs) and hence preclude the priming of CD8^+^ T cells [22]. However, during the process of extracellular release and hydrolysis, ATP can also exert its antitumor function through activating the antitumor immune response, modulating the transition and maturation of DCs to present antigens and migration into TIME via P2X7R and P2Y2R on DCs and other immune cells, stimulating chemotaxis, inducing activation of NLR family pyrin domain-containing protein 3 (NLRP3) inflammasome, triggering intracellular proteolytic transition of pro-interleukin 1β (pro-IL-1β) into IL-1β, and upregulating pro-inflammatory cytokines such as IL-2, IL-12, and interferon γ (IFN-γ), and thus resulting in immunogenic cell death (ICD) with the augment of antitumor immunity [23–25].

### ADO in TIME

Besides ATP, ADO can also involve in tumorigenesis and immunosuppression in TIME. A2AR-deficient mice were observed to have less tendency of tumorigenesis comparing to the wild type controls, given A2AR’s role of protecting tumors from the attack of CTLs, especially in ADO-rich TIME [26]. The similar effect of ADO has also been observed in prostate cancers, in which prostatic acid phosphatase can generate large amounts of ADO to antagonize the impact of tumor-infiltrating lymphocytes and form an immunosuppressive TIME [27]. A2AR agonists can not only control cytokine secretion related to the function of T cells but also endow long-lasting regulation of effector T cells (T_eff_), while A2AR antagonists can synergize with immune checkpoint inhibitors (ICI) to revert tumor immunosuppression [28, 29]. Through A2AR, high amounts of ADO can inhibit proliferation, adhesion, migration, cytotoxicity, and differentiation of T_eff_, for example, suppressing pro-inflammatory cytokine production of IL-2, IFN-γ, and tumor necrosis factor α (TNF-α), downregulating costimulatory receptors such as T-cell receptor (TCR) and CD28, and upregulating immune checkpoints including programmed cell death protein 1 (PD1) and cytotoxic T-lymphocyte associated protein 4 (CTLA4) [30, 31]. Increased ADO reduces the activation of nuclear factor kappa-light-chain-enhancer of activated B cells (NF-κB) in CD4^+^ T cells to modulate the release of a variety of pro-inflammatory cytokines and chemokines [32]. ADO-A2AR signal on the surface of CD4^+^ T cells can upregulate the expression of forkhead box P3 (FOXP3) to support Tregs [33], which can further induce suppression of immune response via pericellular secretion of ADO to favor the M2 polarization of macrophages [34]. Further, ADO-A2AR/A2BR can hinder the differentiation of monocytes into DCs and the DC-mediated function of Th1 and Th17, while impeding their regulation of the differentiation of T_eff_ and upregulating pro-tumorigenic cytokines including IL-6, IL-8, IL-10, transforming growth factor β (TGF-β), and vascular endothelial growth factor (VEGF) [14, 35]. ADO also impedes macrophages-induced phagocytosis, downregulates the release of pro-inflammatory components including IL-12, TNF-α, nitric oxide, superoxide, stimulates pro-tumorigenic factors including IL-10, arginase-1 and VEGF expressed by tumor-associated macrophages (TAM), and increases the expression of VEGF via A2BR and IL-10 via A2AR on myeloid-derived suppressor cells (MDSC) [36–41]. In addition, ADO-A2AR is able to preclude the maturation, activation, proliferation, and cytotoxicity of natural killer (NK) cells to suppress their secretion of pro-inflammatory cytokines including IFN-γ and TNF-α, while the ablation of ADO signaling promotes NK maturation and reduces tumor growth [42]. In neutrophils, ATP and ADO can distinctly function in an autocrine mode where ATP stimulates the oxidative burst and vascular endothelium adhesion for neutrophil activation, whereas ADO reduces tissue injury based on a feedback loop against maintaining consistent inflammatory reaction [43, 44].

### CD39 and CD73 in TIME

Moreover, as the key enzymes to regulate the hydrolysis of ATP, CD39 and CD73 are also essential factors from purinergic pathway to modulate TIME. The expression of CD39 is observed on less than 5% of CD8^+^ T cells and 20–30% of CD4^+^ T cells in human peripheral blood, and is modulated by pro-inflammatory cytokines in oxidative stress and hypoxia [45]. CD39 and ADO can be activated by various stimuli and provoke oxidative stress and the upregulation of cytokine cascades including TGF-β, hypoxia-induced factor 1 (HIF1), IL-6, IL-18, and TNF-α in chronic inflammatory response [44, 46, 47], whereas CD73 is differentially regulated by cytokines including TGF-β, TNF-α, IL-1β, and prostaglandin E2 (PGE2) [48]. CD73 can help tumors evade immunosurveillance via hindering the expansion and function of CTLs and T_H_ cells, which depends on the generated ADO that affects Treg and Th17 cells [44, 48]. In addition, CD73 can regulate T cell homeostasis and memory T cell survival, differentiation, and function in hypoxia milieu. The active enzymatic format of CD39 and CD73 secreted by Tregs can hydrolyze extracellular ATP to ADO within seconds to increase the pericellular ADO level and mediate Treg-induced immunosuppression and anti-inflammatory reaction [12, 49]. In a pancreatic neuroendocrine tumor model, inhibition of purinergic receptors and CD73 harnessed the proliferation of cancer cell, tumor growth, and metastases of cancer stem cells [50]. In DU145 prostate cancer model, exosomes expressing CD39 and CD73 secreted from prostate cancer cells inhibited DC activities, resulting in an immunosuppressive environment to impede the priming and activation of CD8^+^ T cells [51]. Further, CD39/CD73 can delicately regulate the differentiation and function of macrophages and their M2 polarization [44]. Among pro-inflammatory M1-polorized macrophages, downregulation of CD39 and CD73 leads to decrease of ATP degradation and increase of its aggregation, while among M2-polarized macrophages, upregulation of CD39 and CD73 can increase ATP hydrolysis into ADO to promote the production of anti-inflammatory cytokines including IL-10 and IL-1 receptor antagonist (IL-1RA) [44]. Furthermore, MDSCs are also ADO-sensitive for physiological function and express CD39 and CD73 to facilitate tumor growth by inhibiting CTLs in colorectal cancers [15, 52]. Although neutrophils widely express CD39 and CD73, exacerbated activation of neutrophils may be attributed to compromised CD39/CD73 function with upregulated chemotaxis and enhanced vascular endothelium adhesion [44].

## The role of purinergic pathway in radiotherapy (RT)-induced ICD in cancer

### Purinergic pathway and RT-induced ICD

Purinergic pathway is involved in the ICD induced by radiation from multiple layers. RT can trigger cancer cell death followed by elevated extracellular ATP secreted by dying cells, which leads to a variety of complicated downstream cell death processes [53, 54]. Cascades of signaling activate the channels and macropores for ATP penetration and further provoke different manners of cancer cell death including apoptosis mediated by pannexin-1 (PANX-1) channels, necroptosis induced by mixed-lineage kinase domain-like pseudokinase (MLKL) pores, and pyroptosis activated by Gasdermin D (GSDMD) and/or Gasdermin E (GSDME) pores [55]. As a form of mixed cell death, ICD is usually induced by two different types of stimuli. Type I ICD inducers included γ-irradiation, ultraviolet C, and chemotherapy (CT) (e.g. methotrexate, doxorubicin, oxaliplatin), while type II ICD inducers usually refer to hypericin-based photodynamic therapy and oncolytic viruses. Both types of inducers can result in the primary and/or secondary stress of endoplasmic reticulum (ER) that results in apoptosis with caspase-activation and lysosomal exocytosis to finally release damage associated molecular patterns (DAMPs) into TIME [53, 56]. There appears to be an enormously complicated and diverse danger signaling pathways regulating the production of DAMPs in cancer cells. Extracellular DAMPs can then recruit effector cells and initiate tissue repairment and regeneration at the same time [49]. As an essential DAMP, ATP can significantly enhance ICD, which can promote cancer immunogenicity that will effectively stimulate innate and adaptive immune responses. The process involves the recruitment and migration of various immune effector cells, autocrine and/or paracrine of essential cytokines, along with the hydrolysis balance between ATP and ADO modulated by enzymes CD39 and CD73 [53].

### Purinergic signaling in post-RT TIME

**Fig. 2:**
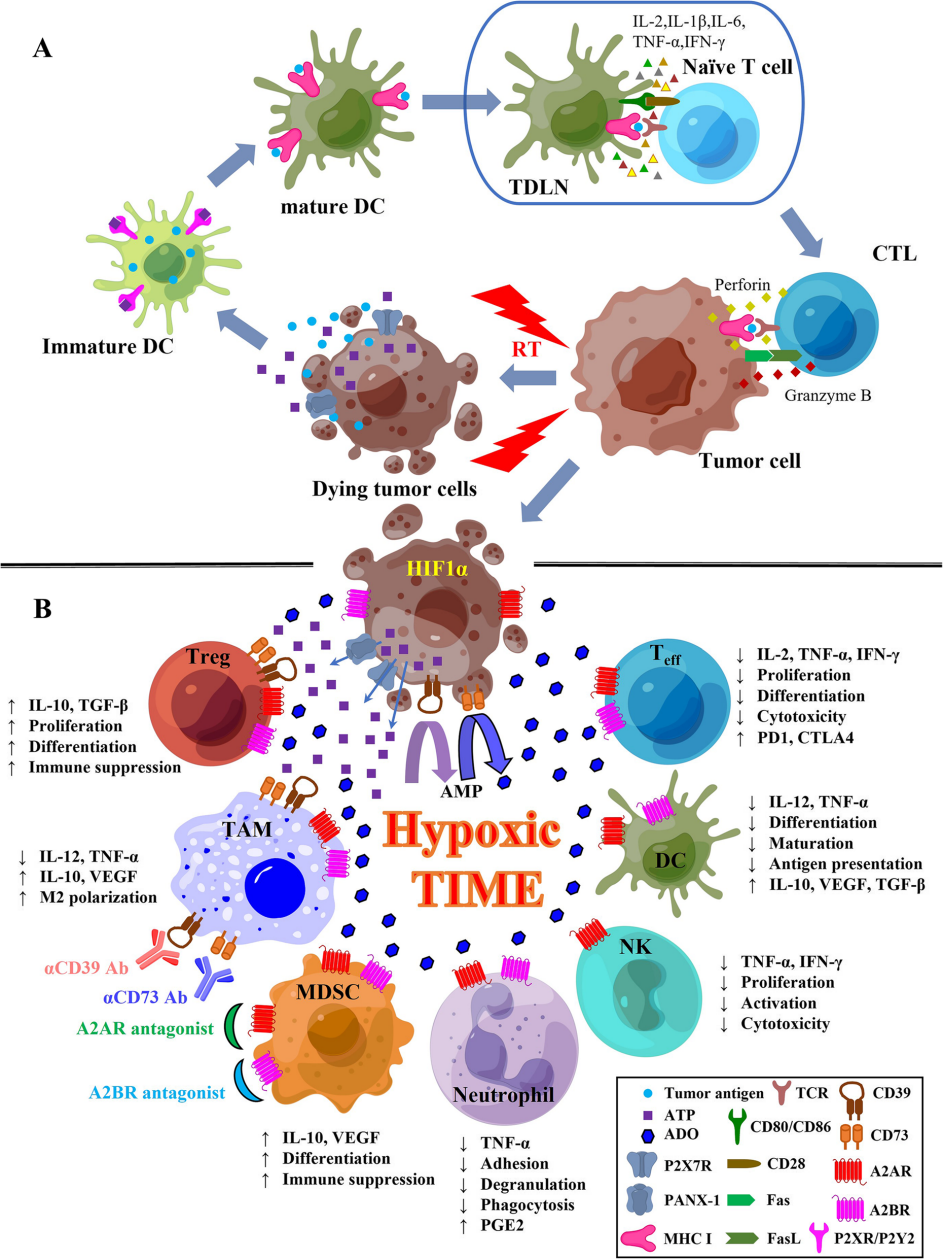
The role of purinergic pathway in RT-induced ICD. **A** ATP in RT-induced T-cell-mediated ICD. Tumor cells release ATP (via cell lysis, channels such as PANX-1, and P2X7R), chemokines, and tumor antigens following RT to recruit immature DCs to TIME. ATP then binds to its receptors (P2XR/P2YR) on immature DCs leading to their activation. These DCs can take up and process tumor antigens released from dying tumor cells to transform into mature DCs. Mature DCs can then migrate to tumor-draining lymph nodes (TDLN), where DCs secret IL-1β, IL-6, TNF-α, and IFN-γ and cross-present tumor antigen peptides by MHC I to stimulate T-cell differentiation to CD8^+^ CTLs. CTLs can then migrate to the tumor site and maybe remote sites to recognize and kill residue tumor cells by releasing perforin, granzyme B, and/or through the interaction of the Fas/FasL signaling. **B** Production of ADO leads to a post-RT immunosuppressive TIME. Hypoxia induced by RT upregulates the expression of HIF1-α, leading to the overexpression of CD39 and CD73 of cancer cells to hydrolyze large amounts of ATP to ADO in TIME. ADO is contributed to an immunosuppressive TIME to enable tumor cells to escape immune-surveillance by suppressing the effector immune components including T_eff_, DCs, NK cells, and neutrophils, while enhancing the activity of immunosuppressive cell subsets including Treg, M2-macrophage, and MDSC, in which pharmacological antagonists against A2AR and/or A2BR as well as blockades inhibiting CD39 and CD73 can reverse ADO-induced immunosuppressive TIME to favor antitumor immunity. A2AR, A2A adenosine receptor; A2BR, A2B adenosine receptor; ADO, adenosine; AMP, adenosine monophosphate; ATP, adenosine triphosphate; CTL, cytotoxic T lymphocyte; CTLA4, cytotoxic T-lymphocyte associated protein 4; DC, dendritic cell; Fas, factor-related apoptosis; FasL, factor-related apoptosis ligand; ICD, immunogenic cell death; IFN-γ, interferon gamma; IL-1β, interleukin-1 beta; IL-2, interleukin-2; IL-6, interleukin-6; IL-10, interleukin-10; IL-12, interleukin-12; MHC I, major histocompatibility complex class I; P2X7R, P2X7 purinergic receptor; PANX-1, pannexin 1; PD1, programmed cell death protein 1; PGE2, prostaglandin E2; RT, radiotherapy; TCR, T-cell receptor; TDLN, tumor-draining lymph node; TGF-β, transforming growth factor beta; TNF-a, tumor necrosis factor alpha; VEGF, vascular endothelial growth factor. Partly created by Figdraw (www.figdraw.com)

RT-induced spatiotemporal pattern of ATP distribution is considered pivotal to form a gradient of extracellular ATP for engendering the chemotactic or DAMP function. For example, ATP was found significantly elevated in conditioned culture media of urothelial cancer cells one hour after irradiation [57]. Irradiation can also cause long-lasting stimulation of extracellular ATP release by glioblastoma cells beyond the acute phase of irradiation-induced cell death [58]. On one hand, ATP released by irradiated dying cells acts like a discoverable “find me” signal for chemoattractant and functions as pro-inflammatory signals for further immune stimulation [54, 59]. After irradiation, an in situ vaccine-like effect was induced in TIME that is enriched with components perturbing the initiation of anti-tumor immune response, while the effect can also be reversed by immunosuppressors in the post-RT TIME. RT efficiency relies on citric acid cycle to increase fatty acid and amino acid oxidation, while radiation-related metabolic change of mitochondrial energy involves the participation of adenosine monophosphate-activated kinase (AMPK) signaling, downstream of which is phosphorylated histone H2A family member X (γH2AX) as a marker of DNA damage [60, 61]. Radiation-induced ATP release can result in the activation of purinergic receptors including P2X7, P2Y2, P2Y6, and P2Y12 [61]. P2X7 is widely expressed on almost all immune cells and promotes IL-1β and IL-18 secretion when activated by ATP, while activation of P2Y2 can promote the recruitment of immature DCs, monocytes, macrophages, and neutrophils [62]. P2Y6 and P2Y12 activation is observed following γH2AX formation by γ-irradiation [63]. In irradiated mice, deficiency of P2YRs in hematopoietic stem/progenitors cells are compromised to maintain hematopoiesis [64]. When RT-induced ICD occurs in TIME, tumor antigens from dying tumor cells can be taken up by mature DCs activated by the interaction of ATP and the above receptors expressed on DC surface, which migrate to tumor-draining lymph nodes (TDLNs) to present/cross-present antigen peptides to T cells that will infiltrate the tumor to recognize and eliminate residue tumor cells [15, 62]. Mature DCs can first secret IL-1β, IL-2, IL-6, TNF-α, and IFN-γ to promote the differentiation of T cells into CD8^+^ subset and then activate those CD8^+^ T cells to CTLs through the cross-presentation of tumor antigen peptides with major histocompatibility complex class I (MHC I). After that, CTLs can proliferate and expand to enhance their antitumor cytotoxicity by increase the secretion of IFN-γ, perforin-1, and granzyme B and/or with a combination of Fas ligand (FasL) with Fas interaction when infiltrate the irradiated tumor site as well as the abscopal sites to induce ICD [62, 65] (Fig. 2A). Mature DCs can also recruit NK cells with IFN-α, IL-2, and IL-12 and enhance their cytotoxicity to secret IFN-γ through the signaling of the C-X3-C motif chemokine ligand 1 (CX3CL1) from DCs and its receptor CX3C chemokine receptor 1 (CX3CR1) on NK cells [66, 67]. On the other hand, however, induced by repetitive stimuli of radiation, ATP may increase the release of caspase-1 and IL-1β via P2Y2 receptor in RT-resistant breast cancer cells and improved the colony-forming and invasion abilities of these cells during inflammatory process, and further promoted tumor growth and invasion while negatively regulating inflammasome activation [68]. Therefore, inhibition of P2X7R radio-sensitized melanoma, while suppression of ATP storage by targeting CD105 / sirtuin 1 (SIRT1) pathway increased radiosensitivity of prostate cancer cells via G2 cell cycle arrest [69, 70].

After RT, intratumoral hypoxia promotes radio-resistance where an immunosuppressive TIME can be further intensified. After ATP is released by dying tumor cells during RT-induced ICD, it can be hydrolyzed to ADO by CD39 and CD73 upregulated by hypoxia-induced HIF1-α in TIME. HIF1-α, the key factor functioning in hypoxia and excessive inflammation in post-RT TIME, can lead to a cascade of immunosuppressive signaling through the proliferation of Treg, the M2 polarization of TAMs, and the enrichment of MDSC [13, 54, 62]. High level of ADO, both expressed in intracellular reservoir and hydrolyzed from extracellular ATP following RT-induced ICD, has been observed in hypoxic TIME, which intervenes with other key signaling pathways to regulate TIME [33, 49]. The aggregated extracellular ADO can inhibit DC and T_eff_, favor the proliferation of Tregs, and polarize suppressive M2 TAMs, which can produce substantial immunosuppression in post-RT high oxidative stress to increase reactive oxygen species (ROS)-induced resistance to ICD [15, 53, 71]. TGF-β expressed by tumor cells stimulates the mammalian target of rapamycin (mTOR) pathway and induces the CD73 production while maintaining the stability of HIF1-α. Wnt pathway upregulates the promoter of CD73 via β-catenin and pro-inflammatory cytokines including TNF-α and hepatocyte growth factor (HGF), with the transcription factor c-Jun / activator protein 1 (AP1) for CD73 transcription accompanied by MAPK signaling pathway, whereas CD73 promoter CpG islands can also be methylated under certain circumstances [72, 73]. Exosomes from cancer may stimulate DCs to express CD73 induced by PGE2 [74]. Regulated by mTOR/HIF1-α pathway, CD39 and CD73 are heterogeneously expressed on MDSCs when exposed to TGF-β, which cooperates with IL-6 to increase the expression of CD73 on Th17 cells during its differentiation [75, 76]. Further, A2AR stimulation by ADO can not only restrict the NK maturation and proliferation, but also their function to produce IFN-γ and TNF-α, which impairs their capacity to eliminate cancer. ADO can also exert various inhibitory effects on neutrophils via A2AR signaling to deteriorate their ability of cytokine secretion (e.g. TNF-α), adhesion, degranulation, and phagocytosis [30, 41] (Fig. 2B).

## Targeting purinergic pathway to enhance RT for cancer treatment

### The summary of novel antitumor therapeutics targeting purinergic signaling pathway

Targeting individual component within purinergic signaling pathway such as ADO, P1 receptors, CD39, or CD73 may be promising with efficacy and safety for cancer immunotherapy. There have been two popular methods to inhibit ADO-induced signaling: (1) direct blockade of the binding of ADO to P1 receptors including A2AR and A2BR to suppress its function, mainly through pharmacological inhibitors/antagonists that are usually orally delivered; and (2) specific blockades of those enzymes including CD39 and CD73 to inhibit ADO production, mainly by specific antibodies that are usually intravenously delivered [41, 77] (Fig. 2B). Nowadays, more than thirty such pharmacological therapeutics including antagonists and antibodies have been or are currently investigated in the clinic for cancer treatment, alone or combined with other therapeutics including CT, RT, and/or ICI, whose benefits have already been reported at the preclinical level in various tumor models [13, 30, 33, 41, 74, 77, 78] (Table 1).

**Table 1:** Blockades of purinergic signaling registered in clinicaltrials.gov

Target	Agent	Pharmaceutical Supplier	Type	Dual-specific	Delivery method	Clinicaltrials.gov Identifier	Phase	Status	Cancer	Single agent	Combination
**A2AR**	Ciforadenant (CPI-444)	Corvus	Antagonist	N/A	Orally	NCT02655822	I	Completed	RCC, mCRPC	YES	Atezolizumab
						NCT04280328	I	Completed	MM	N/A	Daratumumab
						NCT03337698	I/II	Recruiting	NSCLC	N/A	Atezolizumab
	CS3005	CStone Pharmaceuticals	Antagonist	N/A	Orally	NCT04233060	I	Completed	Advanced solid tumors	YES	N/A
	Etrumadenant (AB928)	Arcus Biosciences	Antagonist	A2AR/ A2BR	Orally	NCT03629756	I	Completed	Advanced malignancies	N/A	Zimberelimab
						NCT03719326	I	Completed	TNBC, OC	N/A	PLD, and/or IPI-549, NP
						NCT03720678	I	Completed	mGEC, mCRC	N/A	CT
						NCT03846310	I	Active, not recruiting	non-squamous NSCLC	N/A	CT, and/or anti-PD1 antibody
						NCT04892875	I	Not yet recruiting	Locally advanced head and neck cancers	N/A	RT, CT, and zimberelimab
						NCT03193190	I/II	Active, not recruiting	mPDAC	N/A	Atezolizumab and CT
						NCT03555149	I/II	Recruiting	mCRC	N/A	Atezolizumab and regorafenib
						NCT04381832	I/II	Recruiting	mCRPC	N/A	Zimberelimab, and/or CT; or with AB680 and/or zimberelimab
						NCT04660812	I/II	Recruiting	mCRC	N/A	Zimberelimab and/or CT, bevacizumab, and Regorafenib
						NCT03821246	II	Recruiting	Localized PC prior to radical prostatectomy	N/A	Atezolizumab
						NCT04262856	II	Recruiting	mNSCLC	N/A	Zimberelimab and domvanalimab
						NCT04791839	II	Recruiting	previously ICI-treated NSCLC	N/A	Zimberelimab and domvanalimab
						NCT05024097	II	Recruiting	Rectal cancer	N/A	RT, CT, and zimberelimab
						NCT05335941	II	Not yet recruiting	Previously treated advanced or metastatic MTAP-deficient UC	N/A	Pemetrexed and zimberelimab
	Imaradenant (AZD4635)	AstraZeneca	Antagonist	N/A	Orally	NCT02740985	I	Active, not recruiting	Advanced solid malignancies	YES	Durvalumab, or abiraterone acetate enzalutamide, or docetaxel; with durvalumab and oleclumab
						NCT03980821	I	Completed	Advanced solid malignancies	YES	N/A
						NCT04089553	II	Active, not recruiting	PC	N/A	Durvalumab or oleclumab
						NCT04495179	II	Active, not recruiting	mCRPC	N/A	Durvalumab, ± cabazitaxel
	INCB106385	Incyte	Antagonist	A2AR/ A2BR	Orally	NCT04580485	I	Recruiting	Advanced solid tumors	YES	INCMGA00012
	Inupadenant (EOS100850)	iTeos Therapeutics	Antagonist	N/A	Orally	NCT03873883	I	Recruiting	Adult solid tumors	YES	Pembrolizumab or CT
						NCT05117177	I	Recruiting	Adult solid tumors	YES	N/A
						NCT05060432	I/II	Recruiting	Advanced solid tumors	N/A	EOS-448
	PBF-999	Palo Biopharma	Antagonist	A2AR/ PDE-10	Orally	NCT03786484	I	Recruiting	Advanced solid tumors	YES	N/A
	Preladenant (MK-3814)	Merck	Antagonist	N/A	Orally	NCT03099161	I	Terminated^a^	Advanced solid tumors	YES	Pembrolizumab
	Taminadenant (NIR178/PBF-509)	Palo Biopharma (Novartis)	Antagonist	N/A	Orally	NCT02403193	I	Recruiting	Advanced solid tumors	N/A	KAZ954
						NCT03742349	I	Recruiting	Advanced/relapsed RCC and other malignancies with HIF stablizing mutations	N/A	PDR001 and DFF332
						NCT04237649	I	Recruiting	Advanced solid tumors	N/A	KAZ954
						NCT04895748	I	Recruiting	Advanced/relapsed RCC and other malignancies with HIF stablizing mutations	N/A	PDR001 and DFF332
						NCT03207867	II	Active, not recruiting	Solid tumors and NHL	N/A	PDR001
**A2BR**	PBF-1129	Palo Biopharma	Antagonist	N/A	Orally	NCT03274479	I	Recruiting	Locally advanced/metastatic NSCLC	YES	N/A
						NCT05234307	I	Not yet recruiting	Recurrent/metastatic NSCLC	N/A	Nivolumab
	TT-4	Tarus Therapeutics	Antagonist	N/A	Orally	NCT04976660	I/II	Not yet recruiting	Advanced selected solid tumors	YES	N/A
	TT-702	Teon Therapeutics	Antagonist	N/A	Orally	NCT05272709	I/II	Recruiting	Advanced solid tumors	YES	N/A
**CD39**	ES002023	Elpiscience Biopharma	Antibody	N/A	I.V	NCT05075564	I	Recruiting	Locally advanced or metastatic solid tumors	YES	N/A
	IPH5301	Innate Pharma	Antibody	N/A	I.V	NCT04261075	I	Active, not recruiting	Advanced solid tumors	YES	Durvalumab, ± oleclumab
						NCT05143970	I	Recruiting	Advanced solid tumors	YES	CT and trastuzumab
	PUR001	Purinomia Biotech	Antibody	N/A	I.V	NCT05234853	I	Not yet recruiting	Advanced solid tumors	YES	N/A
	SRF617	Surface Oncology	Antibody	N/A	I.V	NCT04336098	I	Recruiting	Adult solid tumors	YES	CT or pembrolizumab, or both therapies
						NCT05177770	II	Recruiting	mCRPC, PC	N/A	Etrumadenant and zimberelimab
	TTX-030	Trishula Therapeutics (AbbVie)	Antibody	N/A	I.V	NCT03884556	I	Active, not recruiting	Advanced cancers	YES	CT or pembrolizumab
						NCT04306900	I	Recruiting	Advanced cancers	N/A	CT, or budigalimab, or pmebrolizumab, or budigalimab and CT
**CD73**	AK119	Akesobio	Antibody	N/A	I.V	NCT04572152	I	Recruiting	Advanced solid tumors	YES	AK104
						NCT05173792	I	Recruiting	Advanced solid tumors	YES	N/A
	ATG-037	Antengene	Antagonist	N/A	Orally	NCT05205109	I	Not yet recruiting	Locally advanced or metastatic solid tumors	YES	Pembrolizumab
	BMS-986179	BMS	Antibody	N/A	I.V	NCT02754141	I/II	Active, not recruiting	Malignant solid tumor	YES	Nivolumab or rHuPH20
	GS-1423	Gilead Sciences	Antibody	CD73/ TGFβRII	I.V	NCT03954704	I	Terminated^b^	Advanced solid tumors	YES	CT
	HLX23	Henlius Biotech	Antibody	N/A	I.V	NCT04797468	I	Not yet recruiting	Advanced solid tumors	YES	N/A
	IBI325	Innovent Biologics	Antibody	N/A	I.V	NCT05119998	I	Recruiting	Advanced solid tumors	YES	Sintilimab
						NCT05246995	I	Not yet recruiting	Advanced solid tumors	N/A	Sintilimab
	INCA00186	Incyte	Antibody	N/A	I.V	NCT04989387	I	Recruiting	Advanced solid tumors	N/A	INCB106385, ± retifanlimab
	JAB-BX102	Jacobio	Antibody	N/A	I.V	NCT05174585	I/II	Not yet recruiting	Advanced solid tumors	YES	Pembrolizumab
	LY3475070	Eli Lilly	Antagonist	N/A	Orally	NCT04148937	I	Active, not recruiting	Advanced cancers	YES	Pembrolizumab
	Mupadolimab (CPI-006)	Corvus	Antibody	N/A	I.V	NCT03454451	I	Recruiting	Advanced cancers	YES	Ciforadenant or pembrolizumab
	NZV930(SRF373)	Surface Oncology	Antibody	N/A	I.V	NCT03549000	I	Recruiting	Advanced malignancies	YES	PDR001 or NIR178, or both agents
						NCT04237649	I	Recruiting	Advanced solid tumors	N/A	KAZ954
	Oleclumab (MEDI9447)	Medimmune (AstraZeneca)	Antibody	N/A	I.V	NCT02503774	I	Active, not recruiting	Select advanced solid tumors	YES	Durvalumab
						NCT03736473	I	Completed	Advanced solid malignancies	YES	N/A
						NCT03773666	I	Active, not recruiting	Muscle-invasive bladder cancer	N/A	Durvalumab
						NCT03819465	I	Not yet recruiting	Previously untreated NSCLC	N/A	Durvalumab, ± CT
						NCT03381274	I/II	Active, not recruiting	NSCLC	N/A	Osimertinib or AZD4635
						NCT03611556	I/II	Active, not recruiting	metastatic pancreatic cancer	N/A	CT, ± durvalumab
						NCT03616886	I/II	Active, not recruiting	Previously untreated locally recurrent inoperable or metastatic TNBC	N/A	Durvalumab and CT
						NCT03742102	I/II	Recruiting	mTNBC	N/A	Durvalumab and CT
						NCT04068610	I/II	Active, not recruiting	MSS-CRC	N/A	Durvalumab, bevacizumab, and CT
						NCT03267589	II	Completed	Relapsed OC	N/A	Durvalumab, tremililumab, and MEDI0562
						NCT03334617	II	Recruiting	NSCLC	N/A	Durvalumab
						NCT03794544	II	Completed	Resectable NSCLC	N/A	Durvalumab
						NCT03822351	II	Active, not recruiting	NSCLC	N/A	Durvalumab
						NCT03833440	II	Recruiting	ICI-resistant NSCLC	N/A	Durvalumab
						NCT03875573	II	Recruiting	Luminal B breast cancer	N/A	SBRT and durvalumab
						NCT04145193	II	Withdrawn^c^	MSS-CRC	N/A	Durvalumab and CT
						NCT04262375	II	Withdrawn^d^	NSCLC, RCC	N/A	Durvalumab
						NCT04262388	II	Withdrawn^e^	PDSC, NSCLC, HNSC	N/A	Durvalumab
						NCT04668300	II	Recruiting	Recurrent, refractory, or metastatic sarcoma	N/A	Durvalumab
						NCT04940286	II	Recruiting	Resectable/borderline resectable primary pancreatic cancer	N/A	Durvalumab and CT
						NCT05061550	II	Not yet recruiting	Resectable NSCLC	N/A	Durvalumab
						NCT05221840	III	Recruiting	Stage III unresectable NSCLC	N/A	Durvalumab
	ORIC-533	ORIC Pharmaceuticals	Antagonist		Orally	NCT05227144	I	Recruiting	Relapsed or refractory MM	YES	N/A
	Quemliclustat (AB680)	Arcus Biosciences	Antagonist		I.V	NCT04104672	I	Recruiting	Gastrointestinal malignancies	N/A	CT or CT and zimberelimab
						NCT04381832	I/II	Recruiting	mCRPC	N/A	Etrumadenant and/or zimberelimab
						NCT04660812	I/II	Recruiting	mCRPC	N/A	AB928 and zimberelimab
	Sym024	Symphogen	Antibody		I.V	NCT04672434	I	Recruiting	Advanced solid tumors	YES	Sym021
	Uliledlimab (TJ004309)	TRACON Pharmaceuticals	Antibody		I.V	NCT03835949	I	Active, not recruiting	Advanced or metastatic cancer	N/A	Atezolizumab
						NCT04869501	N/A	No longer available	Advanced or metastatic cancer	N/A	Atezolizumab
						NCT04322006	I/II	Recruiting	Advanced solid tumors	YES	Anti-PD1 antibody
						NCT05001347	II	Recruiting	OC and selected solid tumors	YES	N/A

Term list: *AK104* an anti-PD1/CTLA4 bispecific antibody, *Atezolizumab* an anti-PD-L1 antibody, *Bevacizumab* an anti-VEGF antibody, *Budigalimab* an anti-PD1 antibody, *CT* Chemotherapy, *Daratumumab* an anti-CD38 antibody, *DFF332* a small molecule inhibitor for HIF2α, *Domvanalimab* an anti-TIGIT antibody, *Durvalumab* an anti-PD-L1 antibody, *EOS-448* an anti-TIGIT antibody, *HNSC* Head and neck squamous cell carcinoma, *ICI* Immune checkpoint inhibitor, *INCMGA00012* retifanlimab an anti-PD1 antibody, *IPI-549* a PI3K-γ inhibitor, *I.V.* intravenously, *LAG525* ieramilimab an anti-LAG3 antibody, *mCRC* metastatic colorectal cancer, *mCRPC* metastatic castration-resistant prostate cancer, *MEDI0562* an anti-OX40 antibody, *mGEC* metastatic gastroesophageal cancer, *MM* multiple myeloma, *mNSCLC* metastatic non-small-cell lung carcinoma, *mPDAC* metastatic pancreatic ductal adenocarcinoma, *MSS-CRC* metastatic microsatellite-stable colorectal cancer, *mTNBC* metastatic TNBC, *NHL* Non-Hodgkin lymphoma, *Nivolumab* an anti-PD1 antibody, *NP* nanoparticle albumin-bound paclitaxel, *NSCLC* non-small-cell lung carcinoma, *OC* ovarian cancer, *PC* prostate cancer, *PLD* pegylated liposomal doxorubicin, *RCC* renal cell cancer, *rHuPH20* recombinant human hyaluronidase PH20 enzyme, *RT* radiotherapy, *SBRT* stereotactic body radiotherapy, *Sintilimab* an anti-PD1 antibody, *Spartalizumab* Pembrolizumab an anti-PD1 antibody, *PDR001* an anti-PD1 antibody, *Sym021* an anti-PD1 antibody, *TNBC* triple-negative breast cancer, *UC* urothelial carcinoma, *Zimberelimab* an anti-PD1 antibody.

^a^The data did not support study endpoints

^b^The decision to discontinue the study was made based on the totality of the clinical, pharmacokinetic, and pharmacodynamic findings. No safety concerns were observed

^c^Study withdrawn prior to enrollment due to changing standard of care landscape

^d^Overall clinical activity (ORR) for oleclumab + durvalumab is minimal across tumor types and does not support further evaluation of this doublet

^e^Overall clinical activity (ORR) for oleclumab + durvalumab is minimal across tumor types and does not support further evaluation of this doublet

### The rationale for combining RT with inhibition of purinergic pathway to improve cancer therapy

Especially for the combination therapeutic strategy with RT, inhibition of purinergic pathway has its unique essence to enhance the efficacy of RT to treat malignancies. For instance, A2BR antagonist PSB603 or A2BR siRNA increased the efficacy of RT in human lung cancer cells by blocking epidermal growth factor receptor (EGFR) translocation and DNA repair response, and reducing radio-resistance [79]. Pretreatment of PSB603 combined with irradiation also significantly suppressed tumor growth both in vitro and in vivo compared to either single-arm treated group in mouse B16 melanoma model [80]. In addition, only the combination of anti-CD73 antibody and RT could significantly delay subcutaneous tumor growth and suppress the lung metasteses through abscopal effect compared to either single treatment option in murine LuM-1 rectal cancer model. This combination also revealed to enhance the cytotoxicity and IFN-γ production of splenocytes in those treated mice [81]. Similar efficacy was also observed in a mouse breast cancer model, in which CD73 blockade with RT restored cDC1 infiltration of TIME under the condition of suboptimal type I IFN induced by RT. Even without RT-induced type I IFN, CD73 blockade was essential for the rejection of the irradiated tumor and remote tumor control as abscopal effect when combined with a CTLA4 blockade [82]. Further, in the human glioblastoma cell line A172, antagonists or siRNA of A2BR and CD73 promoted γ-irradiation-induced cell death while suppressed γ-irradiation-induced cell migration and actin remodeling [83]. In human pancreatic cancer cell line MIA PaCa-2, knockdown of CD73 using shRNA also re-sensitized the radioresistant cells to irradiation and restored irradiation-induced apoptosis [84]. Currently, there are several clinical trials registered to investigate a combination of inhibition of purinergic pathway, RT, and other therapies to treat cancer: PANTHEoN [A Study of Concurrent Chemoradiation in Combination With or Without PD1 Inhibitor (AB122) A2AR/A2BR Inhibitor AB928 Therapies in Locally Advanced Head and Neck Cancers. Phase I, NCT04892875] and PANTHER [A Phase II Study to Test the Efficacy of AB928 (a Dual Adenosine Receptor Antagonist) and AB122 (a PD1 Checkpoint Inhibitor) in Combination With Short Course Radiotherapy and Consolidation Chemotherapy for Rectal Cancer. Phase II, NCT05024097], in which a dual-specific A2AR/A2BR antagonist, AB928, will be combined with RT, CT, and zimberelimab, an anti-PD1 antibody, to treat head and neck cancer and rectal cancer; as well as Neo-CheckRay (Neo-adjuvant Chemotherapy Combined With Stereotactic Body Radiotherapy to the Primary Tumour ± Durvalumab, ± Oleclumab in Luminal B Breast Cancer. Phase II, NCT03875573), in which an anti-CD73 antibody, oleclumab, will be combined with stereotactic body radiotherapy (SBRT) and an anti- programmed cell death protein ligand 1 (PD-L1) antibody, durvalumab, for the treatment of luminal B breast cancer.

Etrumadenant (AB928) is the first dual A2AR/A2BR antagonist investigated in the clinic. AB928 is developed to inhibit the ADO-induced impairment of lymphocytes (CTLs and NK cells) and myeloid cells (DCs and macrophages) in TIME, mediated by A2AR and A2BR, respectively. AB928 inhibits A2AR and A2BR with similar high potencies [equilibrium binding constant (K_b_) of 1.4 and 2 nM, respectively]. AB928 has already demonstrated a favorable and well-tolerable safety profile with other reagents and exhibits consistent pharmacokinetics (PK) / pharmacodynamics (PD) by oral dosing [85, 86]. AB928 is currently being evaluated in combination with CT, RT, ICI, and targeted therapeutics in several Phase I, I/II, and II clinical trials among multiple malignancies including advanced or metastatic head and neck cancer, lung cancer, colorectal cancer, pancreatic cancer, and prostate cancer (Table 1).

Oleclumab (MEDI9447) is a human IgG1λ monoclonal antibody (mAb) that inhibits the function of CD73, upregulation of which has been shown to increase extracellular ADO level and lead to subsequent immunosuppressive TIME in multiple cancers [87, 88]. Additive antitumor immunity has been reported when oleclumab is combined with other immunotherapeutics such as ICI in preclinical cancer models [89]. Although the phase I clinical trial of oleclumab in combination with durvalumab only provided marginally improved objective response rate (ORR) with a tolerable safety profile in patients with advanced *EGFR*–mutated non-small cell lung cancer (NSCLC) [90], recent results from the randomized phase II COAST trial in stage III NSCLC revealed a promising ORR of 30.0% in oleclumab + durvalumab arm (vs. 17.9% in durvalumab alone arm) with a statistically improved 12-month progression-free survival (PFS) rate (62.6% in oleclumab + durvalumab arm vs. 33.9% in durvalumab alone arm), whereas all-cause grade ≥ 3 treatment-emergent adverse events occurred in 40.7% and 39.4% with durvalumab + oleclumab and durvalumab, respectively [91]. Oleclumab has already been investigated or is going to be examined in various studies combined with CT, SBRT, ICI, and targeted therapeutics in several Phase I, I/II, and II clinical trials among multiple malignancies including breast cancer, lung cancer, colorectal cancer, pancreatic cancer, ovarian cancer, and bladder cancer, as well as a Phase III clinical trial in stage III unresectable NSCLC following the aforementioned successful Phase II COAST trial.(Table 1).

## Future directions

Purinergic pathway is critically involved in a range of pathological processes including cancer. ADO concentration can be significantly elevated in a variety of malignancies, predominantly due to stress-induced ATP release, for example, irradiation, along with the overexpression of ectonucleotidases including CD39 and CD73 that contribute to its hydrolysis. Primarily by binding to A2AR and A2BR, also often overexpressed in the TIME due to hypoxia and inflammation, ADO impedes the activity of protective immune infiltrated effectors including DCs, T_eff_ cells, and NK cells, whereas promotes immunosuppressive components including Tregs, M2-TAMs, and MDSCs. In addition, ADO also stimulates neo vessel formation to further support tumor growth and metastasis. Administration of pharmacological antagonists or antibodies to block purinergic signaling, either by its binding to their receptors or limiting its production, has achieved significant antitumor efficacy in various pre-clinical cancer models, leading to dozens of trials to investigate them in the clinic.

**Fig. 3:**
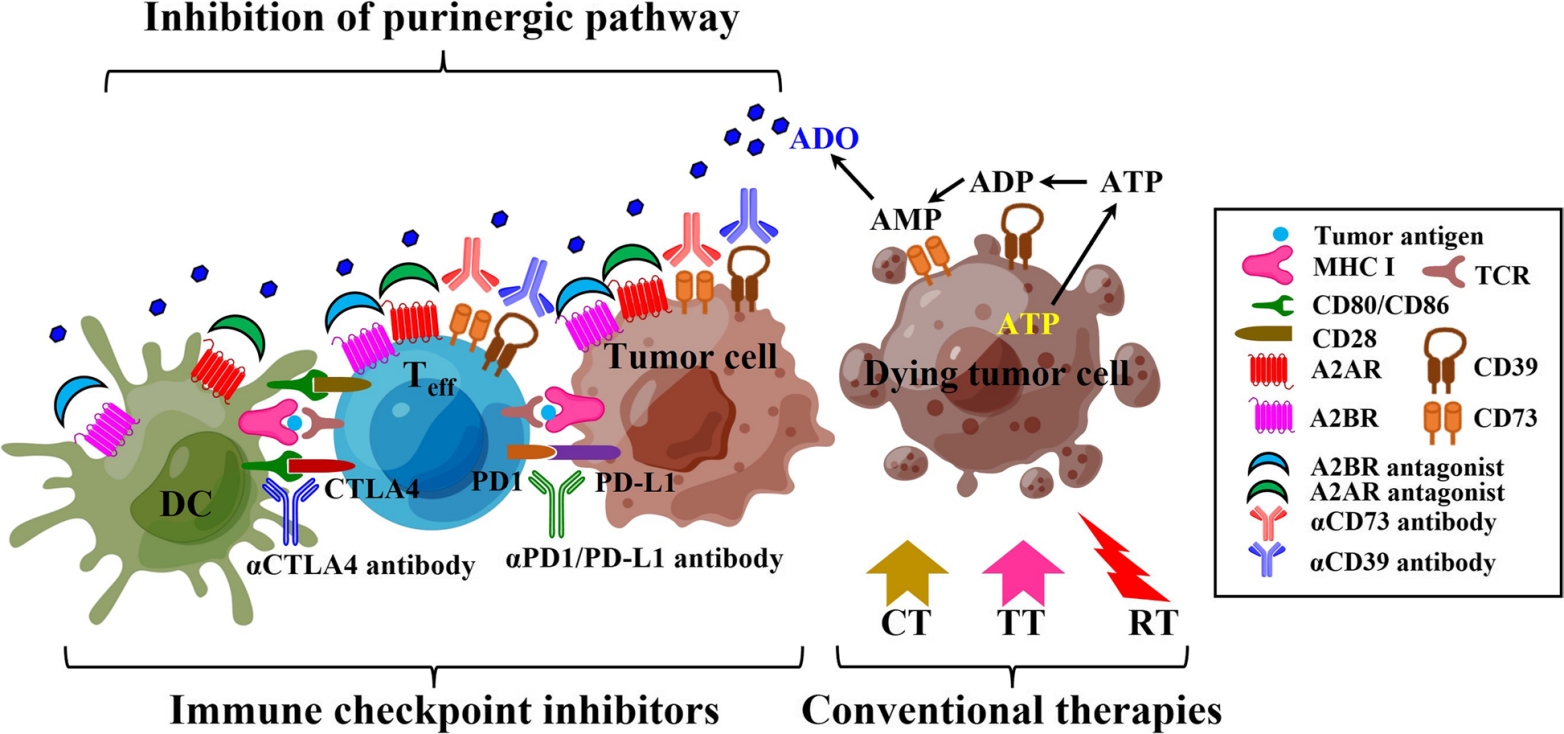
The strategy to combine inhibition of purinergic pathway and other antitumor therapies. Several components within purinergic signaling pathway including A2AR, A2BR, CD39 and CD73 can be targeted together to achieve synergy in antitumor efficacy by modulating both tumor cells and immune cells, for example, DC and T_eff_, by pharmacological antagonist and/or antibodies. In addition, targeting purinergic pathway in combination with other therapies such as ICIs, CT, TT, and RT can also develop potential robust strategies to enhance therapeutic benefit in various cancers, which is currently examined and will be further demonstrated in many important clinical trials. A2AR, A2A adenosine receptor; A2BR, A2B adenosine receptor; ADO, adenosine; ADP, adenosine diphosphate; AMP, adenosine monophosphate; ATP, adenosine triphosphate; CT, chemotherapy; CTLA4, cytotoxic T-lymphocyte associated protein 4; DC, dendritic cell; ICI, immune checkpoint inhibitor; MHC I, major histocompatibility complex class I; PD1, programmed cell death protein 1; PD-L1, programmed cell death protein ligand 1; RT, radiotherapy; TCR, T-cell receptor; T_eff_, effector T cell; TT, targeted therapy. Partly created by Figdraw (www.figdraw.com)

Furthermore, since the synergy of such modulators for purinergic signaling has already been shown with CT or RT that is known to promote ICD, as well as with other immunotherapies such as ICI, increasing numbers of clinical trials combining ADO blockades with ICIs and/or conventional treatment approaches such as RT and CT have been registered for investigation, although blockades of CD39 and CD73 as well as A2AR/A2BR antagonists are also being tested in the clinic as monotherapies. In addition, simultaneous inhibition of adenosine production (e.g. by an anti-CD73 antibody, mupadolimab) and receptor binding (e.g. by an A2AR antagonist, ciforadenant) has also demonstrated its potential synergy, and is under investigation among multiple advanced cancers in the clinic (NCT03454451) (Fig. 3). Despite the novelty of targeting this pathway with aforementioned strategies, an overall understanding of purinergic molecules and their detailed rationales in terms of modulation and effect, autocrine and/or paracrine, positive and negative feedbacks, in RT-induced ICD in TIME are crucial aspects to be further investigated in the future, which also endow pillar effects in downstream reaction and are reflected eventually in the development of innovative efficient combination therapeutic strategies based on RT for cancer treatment.

## Conclusions

In conclusion, purinergic pathway functions as a key factor in RT-induced ICD along with downstream immune responses in TIME. Research data in the combination therapeutic strategies targeting purinergic pathway with RT and/or other emerging immunotherapeutic modalities such as ICIs are accumulating with promising results, which have already led to dozens of clinical trials in advanced and metastatic cancers. Further, extensive and thorough investigation for comprehensive understanding of the rationale, regulation, and modulation of purinergic pathway in TIME and its combination with RT and other novel antitumor therapeutics in larger population and various malignancies is warranted.

## Data Availability

Not applicable.

## Abbreviations

A1R: Adenosine 1 receptor
A2AR: Adenosine 2A receptor
A2BR: Adenosine 2B receptor
A3R: Adenosine 3 receptor
AC: Adenylate cyclase
ADA: Adenosine deaminase
ADO: Adenosine
ADP: Adenosine diphosphate
ALP: Alkaline phosphatase
AMPK: Adenosine monophosphate-activated kinase
ATP: Adenosine triphosphate
cAMP: Cyclic adenosine monophosphate
CT: Chemotherapy
CTL: Cytotoxic T lymphocyte
CTLA4: Cytotoxic T-lymphocyte associated protein 4
CX3CL1: C-X3-C motif chemokine ligand 1
CX3CR1: CX3C chemokine receptor 1
DAMP: Damage-associated molecular pattern
DC: Dendritic cell
EGFR: Epidermal growth factor receptor
ENPP: Ectonucleotide pyrophosphatase / phosphodiesterase
ER: Endoplasmic reticulum
ERK: Extracellular signal-regulated kinase
FasL: Fas ligand
FOXP3: Forkhead box P3
GPCR: G-protein coupled receptor
GSDMD: Gasdermin D
GSDME: Gasdermin E
HGF: Hepatocyte growth factor
HIF1: Hypoxia-induced factor 1
ICD: Immunogenic cell death
ICI: Immune checkpoint inhibitor
IFN-α: Interferon α
IFN-γ: Interferon γ
IL-10: Interleukin 10
IL-12: Interleukin 12
IL-18: Interleukin 18
IL-1β: Interleukin 1β
IL-1RA: IL-1 receptor antagonist
IL-2: Interleukin 2
IL-6: Interleukin 6
IL-8: Interleukin 8
K_b_: Equilibrium binding constant
mAb: Monoclonal antibody
MAPK: Mitogen-activated protein kinase
MDSC: Myeloid-derived suppressor cell
MHC I: Major histocompatibility complex class I
MLKL: Mixed-lineage kinase domain-like pseudokinase
MMP: Matrix metalloproteinase
mTOR: Mammalian target of rapamycin
NF-κB: Nuclear factor kappa-light-chain-enhancer of activated B cells
NK: Natural killer cell
NLRP3: NLR family pyrin domain-containing protein 3
NSCLC: Non-small cell lung cancer
NT: Nucleoside transporter
ORR: Objective response rate
P2XR: P2X receptor
P2YR: P2Y receptor
PANX-1: Pannexin-1
PD: Pharmacodynamics
PD1: Programmed cell death protein 1
PD-L1: Programmed cell death protein ligand 1
PFS: Progression-free survival
PGE2: Prostaglandin E2
PK: Pharmacokinetics
ROS: Reactive oxygen species
RT: Radiotherapy
SBRT: Stereotactic body radiotherapy
shRNA: Short-hairpin RNA
siRNA: Small interfering RNA
SIRT1: Sirtuin 1
TAM: Tumor-associated macrophage
TCR: T-cell receptor
TDLN: Tumor-draining lymph node
T_eff_: Effector T cell
TGF-β: Transforming growth factor β
T_H_: T helper cell
TIME: Tumor immune microenvironment
TNF-α: Tumor necrosis factor α
Treg: Regulatory T cell
UDP: Uridine diphosphate
UTP: Uridine triphosphate
VEGF: Vascular endothelial growth factor
γH2AX: Phosphorylated histone H2A family member X
